# The Role of Tumor Stem Cell Exosomes in Cancer Invasion and Metastasis

**DOI:** 10.3389/fonc.2022.836548

**Published:** 2022-03-08

**Authors:** Kun Liu, Xin Gao, Baoqiang Kang, Yunpeng Liu, Dingding Wang, Yi Wang

**Affiliations:** ^1^ Department of Regenerative Medicine, School of Pharmaceutical Sciences, Jilin University, ChangChun, China; ^2^ Department of Thoracic Surgery, The First Hospital of Jilin University, Changchun, China; ^3^ School of Biosciences and Biopharmaceutics, Guangdong Pharmaceutical University, Guangzhou, China

**Keywords:** cancer stem cells, exosomes, metastasis, tumor, tumor microenvironment

## Abstract

Exosomes are lipid membrane bilayer-encapsulated vesicles secreted by cells into the extracellular space. They carry abundant inclusions (such as nucleic acids, proteins, and lipids) that play pivotal roles in intercellular communication. Tumor stem cells are capable of self-renewal and are crucial for survival, proliferation, drug resistance, metastasis, and recurrence of tumors. The miRNAs (microRNAs) in exosomes have various functions, such as participating in inflammatory response, cell migration, proliferation, apoptosis, autophagy, and epithelial-mesenchymal transition. Tumor stem cells secrete exosomes that act as important messengers involved in various tumor processes and several studies provide increasing evidence supporting the importance of these exosomes in tumor recurrence and metastasis. This review primarily focuses on the production and secretion of exosomes from tumors and tumor stem cells and their effects on cancer progression. Cancer stem cancer derived exosome play an important massager in the tumor microenvironment. It also emphasizes on the study of tumor stem cell exosomes in the light of cancer metastasis and recurrence aiming to provide valuable insights and novel perspectives, which could be beneficial for developing effective diagnostic and treatment strategies.

## Exosomes

Exosomes are extracellular vesicles (EVs) with a double-layer membrane structure without replication ability that all cells can secrete and release externally (1). *In vivo*, exosomes exist in body fluids such as blood, urine, cerebrospinal fluid, pleural fluid, abdominal fluid, saliva, amniotic fluid, breast milk, and semen, and can also be isolated from cell supernatants derived from *in vitro* cell cultures. Exosomes were previously regarded as worthless waste, and recent studies have shown that they play a pivotal role in cell interaction (2, 3). Exosomes can transfer genetic material from donor cells to recipient cells, and are also crucial in cell-to-cell communication, especially remote cell information interaction (4–7). The contents carried by exosomes contain diverse components. Cells can influence another cell through the action of exosomes; in some cases, exosomes enable cells to transfer their genetic material to another cell (8, 9). Exosomes carry a variety of components, including proteins, nucleic acids, amino acids, and metabolites, which may be related to the components and functions of their secretory cells. They are naturally secreted under physiological or pathological conditions (3, 10–12). In physiology, related research on stem cells, tissue regeneration, bone reconstruction, angiogenesis, and other fields has been reported (13–15). In pathology, especially in tumor research, the application of tumor-derived exosomes has been intensively investigated (10, 13, 16, 17). Exosomes play an important role in carcinogenesis. They have been found to participate in many important cancer processes and have the potential to be used as cancer markers. They also drive the key multi-stage tumor process, which provides important therapeutic targets (18–21). *In vitro* studies have shown that exosomes secreted by tumors can induce high cell proliferation (22–24), invasion, metastasis (25, 26), acquired drug resistance (27), angiogenesis (28–31), and the establishment of the pre-metastasis niche (32–34). In addition, exosomes have been used in clinics as therapeutic drugs and biomarkers (35–37). Tumor-derived exosomes play an important role in multiple stages of cancer progression. Cancer stem cells (CSCs) are a subpopulation of tumor cells that contribute to resistance to chemotherapy drugs, metastasis, and recurrence in the development of tumors. Studying the exosomes secreted by CSCs and their anti-tumor clinical applications is of far-reaching significance. This article reviews the role of exosomes secreted by tumor cells in tumorigenesis and development, providing insight into their potential clinical applications.

### Discovery of Exosomes

Exosomes were first discovered in 1983 in vesicles secreted by immature red blood cells of sheep (38, 39). Later, it was discovered that all types of cells can secrete exosomes and can be detected in body fluids. Exosomes have received widespread attention for their important functions, such as changing the extracellular microenvironment and participating in immunity (34). Exosomes contain mRNAs and miRNAs that can mediate cell-to-cell communication (5). Exosomes play an important role in tumor progression as messengers in cellular interactions, homeostasis of the tumor microenvironment, and formation of metastatic foci. Exosomes play an important part in tumor microenvironment as an important messenger with genetic information and proteins during cancer progression.

### Formation of Exosomes

Exosomes are nanometer-sized (diameter = 30–150 nm, average 100 nm) membranous EVs formed by endocytosis (40). They have a double-layer membrane structure and a saucer-like morphology, abundant contents (including nucleic acids, proteins, lipids, etc.), and the ability to participate in information transfer among cells.

Exosomes originate from cells budding inward, forming an early membrane-endosome structure. Early endosomes mature into late endosomes and then accumulate in the lumen to form multivesicular bodies (MVBs), which can also be called multivesicular endosomes (MVEs), which contain many intraluminal vesicles (ILVs). After cells form MVEs, they may be degraded by fusion with auto-phagosomes or lysosomes, or they may be fused with the plasma membrane to release the substances in them. These ILVs are the final exosome formed (40). Exosomes belong to a subgroup of EVs, which are composed of vesicles of different sizes, mainly formed by cells through budding into early endosomes and MVEs to form ILVs, and are released through the fusion of MVEs with the plasma membrane (7, 8, 37).

The composition of exosomes differs depending on the cell source, indicating that tumor-derived exosomes may have specific markers (41). Compared with cell membranes, the phospholipid bilayer of exosomes is rich in lipids, such as cholesterol, ceramide, phosphatidylcholine, phosphatidyl ethanol sphingomyelin, and glycosphingolipid (42). During MVE formation, the surface of exosomes carries various proteins, such as transmembrane proteins, fusion proteins, receptor EGFR, adhesion molecules, integrin, histocompatibility complexes MHC I and II, cytokine protein ESCRT complex protein, and lipid raft-associated protein (43). Different exosomes have conserved components. The most common exosomal marker proteins are integrins and adhesion molecules, mainly CD9, CD63, CD81, MHC, TSG101, and HSC70, which can be used for the identification and sorting of exosomes (9, 37, 44). The main nucleic acids carried by exosomes include miRNA, mRNA, lncRNA, gDNA, and mtDNA (5, 45).

The process of the substances carried by exosomes is not random. The composition is determined by a specific mechanism and can change in response to the tumor microenvironment and stress conditions, thereby affecting the recipient cells (46–51). After secretion, exosomes interact with the recipient cells through different mechanisms, and the receptors or ligands contained in the recipient membrane can activate or stimulate multiple signaling pathways; they can also transfer the contents to the recipient cells through internalization. Exosomes can also be taken up by recipient cells through mechanisms such as phagocytosis and pinocytosis (52). The following mechanisms of exosomes bioactive molecules what influence target calls have been shown: (1) direct stimulate target cells by surface-binding ligands; (2) transfer activated receptors to recipient cells; (3) epigenetic reprogram recipient cells *via* delivery of functional proteins, RNAs and lipids (53). Through these mechanisms, exosomes can alter gene expression and protein translation modification (11).

### Isolation and Identification of Exosomes

It is very important to isolate pure exosomes to study their mechanism of action and application in biomedical science. Exosomes have been successfully isolated by techniques such as super centrifugation, ultrafiltration, chromatography, polymer-based precipitation, and affinity capture of antibody-coupled magnetic beads. In the field of exosome research, there is no unified, simple, feasible, and high-purity separation method. At present, no extraction method can guarantee the content, purity, and biological activity of exosomes at the same time. The appropriate method is chosen based on the topic and experiment. The overspeed centrifugal method is widely used. The advantages and disadvantages of several methods for obtaining exosomes are shown in **Table 1**.

**Table 1:** Summary of isolation methods for exosomes.

Isolation methods	Advantages and disadvantages
Ultracentrifugation	Time consuming. Require specific equipment. Contamination by other Evs. Simple Cost-effective.
Sucrose density gradient ultracentrifugation	Time-consuming, hard to access ultra- centrifugation equipment, change in osmotic environ- ment, co-isolating contaminants. Most studied and most commonly used, easy to handle with simple principle.
Immunoaffinity	Enables continuous mixing and isolation of EVs using immunomagnetic beads. EVs are enriched by immunomagnetic selection and retained as tight aggregates by magnetic force. The retained clusters could be subsequently probed with secondary markers for optical detection. Suitable for different EVs Allows positive and negative selection. Expensive.
Contact-free sorting of EVs	The device consists of a pair of interdigitated transducers to generate standing ultrasound wave to exert differential forces on vesicles of different sizes. During operation, vesicles in an acoustic region experience radiation pressure that is proportional to the vesicle size and move toward the pressure node. Larger vesicles move faster than smaller vesicles, thereby forming differential separation trajectories. By *in situ* tuning of cutoff size, vesicles could be separated with versatile size selection and a good separation yield.
Microfluidic filtering methods for EV isolation and sorting	The device consists of size-selective filters (<1 μm) and capillary guide and is assembled by a magnetic sandwich. A nanoscale lateral displacement array that sorts differentially sized vesicles through displacement trajectories. Due to the differential vesicle trajectories, larger vesicles would be displaced to the right channel (fully bumped) while small vesicles followed a zigzag path. When sorted in the device, fluorescent-labeled EVs confirmed the differential displacement trajectories.
Filtration	Easy. Inexpensive. Suitable for big volumes. Losses during the process. Deform particles.
Size-exclusion chromatography	Suitable for different EVs Inexpensive. Higher purity. Not suitable for big volumes Time-consuming. Require specific equipment.
Microfluidics technology	Good results with small amounts. Fast and simple. Expensive.
Commercial kits	Easy Simple. Expensive. Long incubation periods. Long incubation periods.

The appropriate method is chosen based on the topic and experiment. The overspeed centrifugal method is widely used. Isolation exosome instruments apply to characterization techniques are shown in **Table 2**.

**Table 2:** Summary of exosome characterization techniques.

Methods	Instrument	Description
Morphological	SEM	Provides three-dimensional surface information.
	TEM	Superior image resolution and can be used with immunogold labeling to provide molecular characterization.
	cryo-EM	Enables analysis of EVs morphology without extensive processing.
	AFM	Provide information on both surface topology and local material properties.
Size	NTA( Nanoparticle tracking analysis )	Tracks individual vesicle scattering over time, as they diffuse and scatter under light illumination then it could determine vesicle concentration and size distribution. Analysis of size and concentration. Fast and easy. Low specificity for same size particles.
	DLS	Measures bulk scattered light from EVs as the vesicles undergo continuous Brownian motion. The dynamic information on the vesicles is derived from an autocorrelation of the scattered intensity and could be used to determine vesicle size. As the original size distribution measured by DLS is intensity-weighted, the data is dominated by large vesicles. It is affected by the color, electrical, magnetic and other physical and chemical properties of the measured substance, and is very sensitive to dust and impurities.
	TRPS (Tunable resistive pulse sensing)	Two fluidic reservoirs, each connected to an electrode, are separated by a membrane with a pore. The ionic current between reservoirs is then measured. When a EV passes through the pore, it blocks the current flow, leading to a transient current decrease.
	SEA (Single EV analysis)	EVs are biotinylated and captured on a flat surface coated with neutravidin (Av). EVs are then stained with fluorescent antibodies and imaged. Subsequently, fluorophores are quenched, and the staining process is repeated for a different set of markers.
	SP-IRIS	The coherent light formed by the substrate and the particle is imaged, and the size of the nanoparticles is directly calculated by the brightness after imaging. Sp-iris technology has the advantages of high precision, high sensitivity and single exosome fluorescence imaging.
Surface marker	Flow cytometry -Small particle flow cytometry	Highly sensitive flow cytometry instrument, termed vesicle flow cytometry. Fluorescent intensity from liposomes, labeled with di-8-ANEPPS, were calibrated for the vesicle diameter. Analysis of size, count and surface protein expression. Commonly found in facilities. Size limitation of conventional flow cytometers
	Western blot	Conventional EV protein analysis. EV protein lysate is separated by SDS−PAGE, before being transferred over to a membrane for immunoblotting of specific EV protein targets (e.g., CD81, TSG101, CD9 and CD63). Analysis of protein components. Technique commonly performed in several laboratories. Time consuming processing.
	ELISA (Enzyme-linked immuno- sorbent assay)	In the specific “sandwich” configuration, vesicles or lysates could be applied to a solid support that has been pretreated with an immobilized capturing antibody. Captured vesicle targets are then exposed to a detecting target antibody.
New Technologies for Analysis of EVs	Micronuclear magnetic resonance	(a) Assay schematics to maximize magnetic nanoparticle (NMP) binding onto EVs. A two-step bio- orthogonal click chemistry was used to label EVs with MNPs. (b) The microfluidic system for on-chip detection of circulating EVs is designed to detect MNP-targeted vesicles, concentrate MNP-tagged vesicles (while removing unbound MNPs), and provide in-line NMR detection.
	Surface plasmon resonance	(a) The nPLEX sensing is based on transmission SPR through periodic nanohole arrays. The hole diameter is 200 nm with a periodicity of 450 nm. The structure was patterned in a gold film (200 nm thick) deposited on a glass substrate. (b) Finite-difference time- domain simulation shows the enhanced electromagnetic fields tightly confined near a periodic nanohole surface. The field distribution overlaps with the size of EVs captured onto the sensing surface, maximizing the detection sensitivity. (c) The sensing array can be integrated with multichannel microfluidics for independent and parallel analyses. (d) Assay schematic of changes in transmission spectra showing EV detection. The gold surface is prefunctionalized by a layer of polyethylene glycol (PEG), and antibody conjugation and specific EV binding were monitored by transmission spectral shifts as measured by sensor. (e) In comparison to gold standard methods, the nPLEX assay demonstrated excellent sensitivity, more sensitive than Western blotting and chemiluminescence ELISA, respectively. (f) Correlation between nPLEX and ELISA measurements. The marker protein expression level was determined by normalizing the marker signal with that of anti-CD63, which accounted for variation in exosomal counts across samples.
	Electrochemical detection	EVs are captured on magnetic beads directly in plasma and labeled with HRP enzyme for electrochemical detection. The magnetic beads are coated with antibodies against CD63(an enriched surface marker in exosomes).
	ExoScreen technology	This proximity assay requires two types of immunobeads: (1) donor beads, which are excited at 680 nm to release singlet oxygen, and (2) acceptor beads, which can be only excited by the released singlet oxygen when they are situated within 200 nm away from the donor beads. (b) Assay workflow. Biological samples are first treated with biotinylated antibodies and acceptor beads conjugated with a second antibody. Streptavidin-coated donor beads were then added to complete the proximity assay for data acquisition. (c) Correlation between ExoScreen measurements for CD9 positive EVs, CD63 positive EVs, or CD63/CD9 double-positive EVs and EV protein concentration in a dilution series. The addition of biotinylated antibodies and acceptor beads conjugated antibodies is denoted “bCD9/aCD9” or “bCD63/aCD63”.

## Cancer Stem Cells

CSCs, also known as tumor-initiating cells, are cells in tumor tissues that have the ability to self-renew and produce heterogeneous tumor cells (54, 55). CSCs were first discovered in leukemia in 1997, were subsequently isolated from a variety of solid tumors, and presumed to be the clonal core of the tumor, playing an important role in multiple stages of tumor progression (56, 57). CSCs have the ability of retro-differentiation, self-replenishment, and self-renewal in tumor tissues, and have potential migration and drug resistance characteristics. They are considered triggers of carcinogenesis that promote cancer progression, spread, metastasis, drug resistance, and recurrence (56, 58, 59). Under the regulation of the tumor microenvironment (TME), the tumor stem cell population changes dynamically, and this change may achieve tumor activity through communication between the tumor and the CSCs. As an important messenger in the TME, exosomes secreted by tumor stem cells play a pivotal role in the dynamic regulation of tumor stem cell populations.

### Origin of Cancer Stem Cells

CSCs are a subpopulation of cells in tumors. After xenotransplantation into immunodeficient animal hosts in *in vivo* experiments, this subpopulation has higher tumorigenicity than tumor cells at a lower cell concentration (56). This indicates that this subgroup of cells has a higher dividing ability, and their daughter cells are self-renewing and highly tumorigenic (60). Subgroups of such cells have also been found in solid tumors (61, 62). Cancer stem cell subgroups are characterized by low content (0.01–2%) and high tumorigenicity (54). A relatively small number of cancer stem cells with self-renewal ability can produce a large number of progeny cells that maintain limited proliferation ability (63). The small population of tumor cells with tumorigenicity expresses some specific antigen markers on the surface that are not expressed in a large population of cells. There are differences between different malignant tumors, and even between individual tumor cells of the same histology (54). Therefore, in the same TME, CSCs and tumor cells are at different stages of differentiation.

There are several hypotheses about the origin of cancer stem cells: (1) some genes of adult stem cells are mutated, their genome stability is reduced, and their cancer-causing mutation induction and self-renewal ability are excessively enhanced, so that they evolve into CSCs (64–66); (2) some genes of targeted progenitor cells have mutations, which enhance their limited self-renewal ability, and eventually evolve into CSCs (67); (3) differentiated non-stem cells use certain regulatory mechanisms, such as endothelial-mesenchymal transition (EMT), obtain the characteristics of stem cells and, as gene mutations occur, they are finally transformed into CSCs (64, 68, 69); (4) inflammatory cytokines may induce the formation of CSCs (70). The origin of CSCs is a multi-factor induction and multistep outcome. There is now a general consensus that differences exist between different malignant tumors and even between individual tumors of the same tissue. The phenotype of CSCs is complex; CSCs in tumors have the same genes, but there are multiple CSC subgroups in each tumor, and each CSC has its own unique biological characteristics (71). Tumor-forming CSCs play a significant role in tumor recurrence during the multi-step carcinogenesis of tumors. Therefore, the signal changes of CSCs in multi-stage tumor progression, especially in the recurrence stage after metastasis, are worthy of in-depth study.

### Characteristics of Cancer Stem Cells

CSCs account for a very low proportion of solid tumor cells, and it is difficult to detect and identify them histologically (72). At present, there are many methods for their identification; serum-free culture into balls, labeling specific surface markers (73), *in vivo* limiting-dilution tumorigenicity assays in immunocompromised mice (74), etc. However, it is still very difficult to create a definitive classification of CSC subgroups (60). Therefore, the sorting of CSC subpopulations is based on the combination of tumor surface-specific antigen sorting and other functional analyses (such as functional recognition) and other screening conditions to establish CSC subpopulations. Cancer cells display diverse phenotypes within a certain period of time. Cell surface markers have been used in a variety of tumors to separate different tumor cell subgroups with different biological characteristics. Commonly used surface antigen markers, such as CD133 antigen, have been used for lung cancer (75–79), brain cancer (80), colon cancer (81, 82), and prostate cancer (83). The isolated cells have strong tumorigenic ability and stem cell characteristics, such as stem cell/progenitor cell markers of normal tissue (84), kidney stem cells (85), prostate stem cells (86), fetal neural stem cells (87), etc. The isolation of CSC subgroups is conducive to accurately studying signal transduction changes.

There are many forms of intratumorally heterogeneous tumor subgroups, showing obvious inter- and intra-tumor diversity (88). The existence of CSC subsets drives tumorigenesis and tumor metastasis (58). The results of exome and whole-genome sequencing indicate that most tumors have at least two driver mutations (89). Heterogeneous tumor cells produced by differentiation and proliferation can be transferred to a variety of tissues, causing the destruction and abnormal functions of normal tissues and organs. These abnormalities may lead to the appearance of different subgroups within the tumor, which have different combinations of dominant genetic susceptibility in different TMEs. In the multi-step process of tumor development and recurrence, signal transduction plays an important role in adaption to the TME in heterogeneous CSC subsets with superior susceptibility combinations. Therefore, studying CSCs in tumors can further elucidate the genesis of their heterogeneity and metastasis, which provides insight and inspiration for the design of novel intervention drugs targeting tumor suppressor signals.

### Clinical Characteristics of CSCs

In-depth CSC research has shown that CSCs participate in metastatic formation, natural resistance to chemotherapy and radiotherapy, and cancer recurrence.

In clinical settings, the limitations of traditional anti-cancer therapies have been attributed to the targeting of the large non-CSC populations in tumors, rather than eliminating the rare subpopulations of CSCs (90–92). The difference between CSCs and non-CSCs could be mainly attributed to the process of EMT (93). EMT changes the heritable phenotype of cancer cells by employing epigenetic modifications rather than by introducing new genetic changes. As the EMT program is activated, cancer cells lose many epithelial cell characteristics, including epithelial cell connections and apical-basal polarity, and acquire mesenchymal characteristics, such as elongated, fibroblast-like morphology and increased migration and invasion ability (94). In some cancers, only tumor cells in the CSC-enriched subpopulation exhibit EMT-activated characteristics (95, 96). It is worth noting that in some experimental cancer models, the forced induction of EMT by epithelial tumor cells increases their ability to initiate tumors (95, 97, 98). These CSCs activate a genetically determined morphogenesis program. Normal CSCs reside in the CSC niche, maintain their stem cell status, and control their self-renewal and differentiation. The TME is also involved in metastasis through EMT, which leads to the spread and metastasis of the tumor (99).

Molecular targeted therapy for acquired drug resistance remains a major challenge in cancer treatment. Drug resistance may be the key to improving the effectiveness of targeted therapies for cancer patients. The dormant or slowly circulating subgroups of tumors keep being viable under treatment. These cells do not exhibit typical resistance-driven changes. The drug-resistant phenotype is temporary and can be reversed after drug removal. Tumors can relapse if treatment is stopped or acquired drug resistance is caused by continued treatment (100). CSCs are important factors in tumor drug resistance. Their mechanism of action includes pumping drugs out of the cell through the high expression of ABC transporters, high expression of anti-apoptotic genes, abnormal DNA damage repair mechanism, increased telomerase activity, self-renewal ability, and general quiescent phase, making drugs resistant to targeting the proliferation cycle (101). An important characteristic of CSCs is the activation of the EMT, during which activated tumor cells are resistant to many types of therapeutic drugs (102–104). Exosomes, as messengers of drug resistance in cancer stem cells under treatment, play a central role in the information transfer of the EMT effect of CSCs.

In the cascade of tumor invasion and metastasis, the most complicated process occurs after the metastatic tumor cells reach the parenchymal tissue, proliferate and form clones in the new environment, and finally form tumor masses. As the new tissue environment cannot provide the migrating tumor cells with the microenvironment of the primary tumor, metastatic tumor cells lacking various growth factors usually die quickly or survive for a period of time as micro-metastases in the form of single cells or small clusters of cells. Considering the tumor-initiating ability of CSCs, active CSCs are prone to form multiple micro-metastases in the body, which are difficult to detect in clinical settings and have the potential to develop or re-develop into new tumor mass precursors. These micro-metastases, which are widely distributed in the patient’s tissues, eventually cause tumor recurrence. In the cascade of tumor invasion and metastasis, early steps result in a very high success rates, but the success rate of colonization, that is, metastasis and recurrence, is very low. The invasion–metastasis cascade involves the migration of tumor cells from the primary tumor to potential sites of metastasis. This phenomenon depends on a series of intricately orchestrated and distinct physiological steps. In the initial step, the EMT occurs *in situ* and the tumor cells break through the basement membrane. Subsequently, these cells enter the blood or the lymphatic circulation and are transported to the distal parts of the body. The tumor cells then stay and penetrate the capillary/lymphatic walls to form occult micro-metastases at the metastatic sites. Some micro-metastases may gain the ability to form micro-clones at the metastatic sites as tumor cells can clone the metastatic foci, they often acquire the ability to form tiny clones at the metastatic sites, thereby, facilitating metastases. In this way the entire process is eventually amplified.

Therefore, when the metastatic cells and their cloned cells form a detectable mass, micro-metastases have already spread across multiple tissues in the body. Although the tumor cells in these micro-metastases have been in a state of growth quiescence for a long time, the isolated tumor cells still have the ability to proliferate *in vitro* and form new tumors (105). As these newly disseminated tumor cells have the same origin, the micro-metastases formed by them have similar genetic backgrounds. Each cell of the secondary micro-metastases has the ability to form clones. These clonal metastatic cells in micro-metastases drive the micro-metastases to quickly grow into clinically detectable metastases and ultimately lead to tumor recurrence. In-depth research on the mechanism of CSCs in the recurrence process is expected to produce new treatment strategies aimed at eradicating CSCs and may lead to a better prognosis.

## The Role of CSC Exosomes in Cancer Metastasis

As carriers, exosomes play an important role in mediating cell communication and material exchange between CSCs and tumor cells and other cells in the microenvironment, regulating processes such as tumor growth metastasis, drug resistance, EMT, angiogenesis, and immune escape, by transporting tumor-related mRNA, miRNA, proteins, etc. In the TME, exosomes secreted by tumor cells are different from those secreted by normal tissue cells (46). Tumors are complex tissues that rely on communication between different cell types, and CSCs can secrete exosomes, participate in the construction of the TME, and maintain the self-renewal of CSCs and the biological behavior of the tumor. Exosomes secreted by tumor cells increase in the TEM, which is typically acidic (the pH shifts from 7.4 to 6.5, which is typical for tumors), which is associated with the formation of malignant tumor phenotypes (106). As information carriers, exosomes play an important role in cell communication locally and remotely, participate in the mutual transformation between non-CSCs and CSCs, and maintain the dynamic balance of CSCs. These exosomes mediate metastasis to organs by regulating the pre-metastatic microenvironment through different pathways, contains the induction of phenotypic changes and cell differentiation, incorporation of different supporting mesenchymal cells, upregulation of pro-inflammatory gene expression, and induction of an immunosuppressive state. However, exosomes may mediate the re-awakening of dormant ecotopes instead of pre-metastatic ecotone formation (107). Growing evidence indicates that stable microvasculature constitutes a dormant niche, whereas sprouting neovasculature sparks micrometastatic outgrowth (108). Exosomes, especially secreted by CSCs play an important role in cancer progression, and the carried miRNA is considered an important molecular marker for tumor diagnosis and prognosis, especially in lung cancer patients (109). Targeting signal pathways regulated by exosomes could act on CSCs to inhibit the occurrence and development of tumors, which has become a hot topic in recent years.

### The Messenger Role of CSC Exosomes in the TEM

CSCs are non-quiescent and dynamically changing solid cell populations. The actual process of CSCs driving tumor progression is complicated; as the tumor progresses, the tumor genome becomes unstable, and the rate at which each generation of cells acquires mutations continues to increase. Therefore, there are genetically heterogeneous subclones in each tumor cell cluster. Accumulated genetic heterogeneity in a specific cell cluster results in different subclones carrying different genetic phenotypic changes. The heterogeneity of CSCs determines the homeostasis of CSCs in a given TEM. The interaction between CSCs and the microenvironment is a key factor in tumor development. Considering the dynamic conversion mechanism of CSCs and non-CSCs as the starting point for research is of great significance to the information transmission of CSCs (110).

CSCs need signal feedback from the TEM to maintain their stemness, regulate the balance between self-renewal and differentiation, and avoid being depleted. Cell communication and substance exchanges among CSCs, tumor cells, and other cells in the TEM are vital for maintaining their dynamic balance. CSCs can influence the surrounding stromal cells through cell-to-cell contact or paracrine signaling molecules. These signals not only recruit cells to the microenvironment, but also change the function and activity of stromal cells. Some differentiated tumor cells (non-CSCs) have lost the characteristics of stem cells and can regain their stem phenotype through dedifferentiation or reprogramming. CSCs and non-CSCs are in a dynamic balance between differentiation and dedifferentiation. This information exchange between cells and the extracellular matrix maintains the stemness of CSCs and promotes tumor survival and development. The interaction between CSCs and their microenvironment is conducive to the maintenance of homeostasis. Exosomes, as information transmitters between cells in the microenvironment of CSCs, play an irreplaceable messenger role.

### Tumor Metastasis and EMT

The first step in the localized invasion of tumor cell metastasis is the phenotypic transformation of primary tumor cells. To obtain mobility and invasion capabilities, tumor cells lose their epithelial cell phenotype and undergo EMT. EMT refers to epithelial cells that have lost intercellular adhesion and lack motility differentiation characteristics, thus have the characteristics of mesenchymal cells. This process is considered to be the basis of morphogenesis and is involved in the formation of tissues and organs during animal embryogenesis (111). A similar process occurs during wound healing, indicating that EMT is also important in adulthood (94). Tumor cells migrate and become motile through EMT, which promotes their invasion (112). Epithelial cells undergo continuous cell activities during the EMT process, including loss of top-bottom polarity structure, destruction of cell-to-cell connections, cytoskeleton remodeling, changing cell morphology, and eventually showing mesenchymal and invasive phenotypes, thereby causing increased cell motility and degradation of the extracellular matrix (113). Most malignant tumors are epithelial tumors. The malignant tumor cells that undergo EMT will gain enhanced migration and invasion abilities, invade the surrounding extracellular matrix, and eventually metastasize to distant sites. EMT plays an important role in the invasive phenotype of colon cancer, thyroid cancer, and breast cancer (114). Once the tumor cells begin to undergo EMT, their phenotype changes and they acquire mobility and the ability to invade adjacent cells, which can promote tumor metastasis. The EMT effect under physiological and pathological conditions not only changes cell morphology and mobility, but also changes the cell gene expression profile. In human tumors, the progression of sarcoma carcinosarcomas is related to EMT; most cancers have partial EMT features and express both epithelial and mesenchymal markers (115). Xenotransplantation experiments *in vivo* have shown that EMT is associated with tumor metastasis *in vivo* (116). EMT-TFs are not only involved in migration and invasion, but also in inhibiting cellular senescence and apoptosis as well as attenuating cell cycle progression and resistance to radiotherapy and chemotherapy (104). The EMT effect of tumor pathogenesis has been increasingly reported to be related to the local infiltration and metastasis of tumor lesions (111).

EMT cells have the ability to resist apoptosis, chemotherapy, and immunotherapy. EMT induces immune tolerance and causes malignant tumor cells to evade immune surveillance. An increasing number of studies have shown that EMT is related to CSC characteristics (103). In the pathogenesis of cancer, tumor cells receive signals transmitted by exosomes secreted by other tumor cells, and induce changes in their phenotype, so that they can acquire the ability of cell proliferation, migration, and invasion. The nuclear driver of EMT has growth inhibition and stimulation functions in different microenvironments. EMT participates in multiple signaling pathways involving multiple related proteins and their transcription. Non-coding RNA, differential splicing, translation, and post-translational control mainly participate in the regulation of EMT (94). Exosomes are rich in small non-coding RNAs or miRNAs, which are now widely regarded as effective modifiers of gene expression, enabling cells to respond quickly to new environments, and are directly or indirectly related to EMT-related miRNAs (117). The miR-200 family has been shown to be downregulated in normal human and mouse mammary stem and progenitor cells (118). EMT is associated with the loss of p53 function. miRNAs are important mediators of p53 to regulate EMT and are related to the expression of the tumor stem cell phenotype of tumor cells and affect tumor proliferation and invasion (119, 120). Therefore, p53 transactivates miR‐200c through direct binding to the miR‐200c promoter. Loss of p53 cells leads to decreased expression of miRNA and activates the EMT programme, accompanied by stemness properties cells increased (121). Tumor cells secrete exosomes containing the miR-200 family, which play an important role in the regulation of the tumor microenvironment in tumor metastasis. As messengers of information exchange between cells, tumor stem cell exosomes are loaded with these non-coding RNAs, which act on the inhibition or expression of EMT-TF and reflecting the important role of EMT in the cancer process, which has research significance. Exosomes take up miRNAs in cancers plays important function in TEM. The cell interaction of exosome miRNA in cancers function shown in **Table 3**.

**Table 3:** Cell interaction of exosome miRNA in cancers.

miRNA	Donor cell	Recipient cell	Target gene	Disease	Function	Reference
miR-25-3p	CRC	EC	KLF4 and KLF2	CRC	metastatic	(29)
miR-1247-3p	HCC	FIB	B4GALT3	LUC	metastasis	(25)
miR-423-5p	GC patients’ serum	GC	SUFU	GC	proliferation and migration	(23)
miR141	lung cancer patients’ serum	LUC cell and EC	GAX	LUC	angiogenesis and malignant	(122)
miR103	HCC	EC	VE-Cad, p120 and ZO1	HCC	migration	(123)
miR-1246	BC	HMLE	CCNG2	BC	migration and chemoresistance	(22)
miR-19b-3p	CCRCC CSC	CCRCC	PTEN	LUC	EMT	(124)
miR-210-3p	LUC CSC	LUC	FGFRL1	LUC	metastatic	(125)
miR21, miR-100-5p, miR-21-5p and miR-139-5p	Pca cells and CSCs	FIB	MMPs -2, -9 and -13 and RANKL	PCa	metastatic	(126)
miR155	PDAC cells resistance gemcitabine	PDAC	TP53INP1	PDAC	chemoresistance	(27)

FIB, fibroblasts; EC, endothelial; CRC, colorectal cancer; HCC, hepatocellular carcinoma; CCRCC, Clear cell renal cell carcinoma; CSCs, cancer stem cells; GAX, homeobox gene; GC, gastric cancer; FGFRL1, fibroblast growth factor receptor-like 1; Pca, prostate cancer; VE-Cad, VE-Cadherin; p120, p120-catenin; ZO1, zonula occludens 1; PDAC, pancreatic ductal adenocarcinoma; MMPs, metalloproteinases; BC, breast cancer; CCNG2, Cyclin-G2; LUC, lung cancer.

EMT is closely associated with cancer progression (112, 127). While epithelial cells undergo EMT, the dynamic changes in cell morphology from the epithelial to mesenchymal phenotype are covered, and epithelial cells transform into migratory and invasive cells. When tumor cells have an EMT effect, not only does the morphology change, but the gene expression profile also changes. Cadherin and vimentin are EMT-TFs, and the stimulating signal pathways are stimulated by transcriptional reprogramming and thus transformed into a regulatory network (128). The expression of epithelial cell markers (E-cadherin and cytokeratin) is inhibited, and the expression of a mesenchymal cytoskeleton intermediate fiber component (vimentin) is induced, which is a prerequisite for the progression of malignant tumors. The cytoplasmic domain of cadherin interacts with a catenin-based complex, which binds to the actin cytoskeleton to regulate adhesion-dependent signal transduction. Loss of function may lead to cancer progression through increased proliferation, invasion, and/or metastasis (94). Tumor-derived exosomes are involved in the EMT regulation process, play a role in intercellular signal transduction in the tumor microenvironment, and regulate recipient cells (129–131).

### Tumor Recurrence and Mesenchymal-Epithelial Transformation (MET)

EMT is an abnormal trans-differentiation program induced by exogenous transcription factors (transforming growth factor, fibroblast growth factor), key signaling pathways (the Hh, notch, or NF-κB signaling pathways), and cytokines in the TME. Extracellular signal by exosome mediated stimulation promotes the overexpression of EMT transcription factors, which trigger the EMT by suppressing epithelial phenotypes, enhancing mesenchymal properties, and inducing the degradation of the basement membrane and extracellular matrix (132). The phenotype induced by EMT is reversible, causing the tumor to acquire a highly malignant growth state. When the tumor cells have completed the process of invasion and metastasis, they have to go through the process of MET to restore the epithelial-like cell phenotype. This reversible process indicates that EMT is usually triggered by microenvironmental signals in which tumor cells are located. Exosome changes its messages to guide tumor cells changing. Tumor cells at the pre-aggressive edge of the primary tumor can obtain heterogeneous signals from the adjacent reactive matrix, which is gradually formed during tumorigenesis and tumor development. Once the tumor cells leave the primary tumor and migrate to the distal end, the interstitial microenvironment that they are located in stops to release EMT-induced signals continuously, and some tumor cells undergo MET conversion and restore the phenotype of their primary tumor. Tumor cells that restore the epithelial phenotype clone a large number of them after interacting with the cells of the tumor microenvironment by exosomes, and finally the tumor recurs after the metastasis is complete.

**Figure 1:**
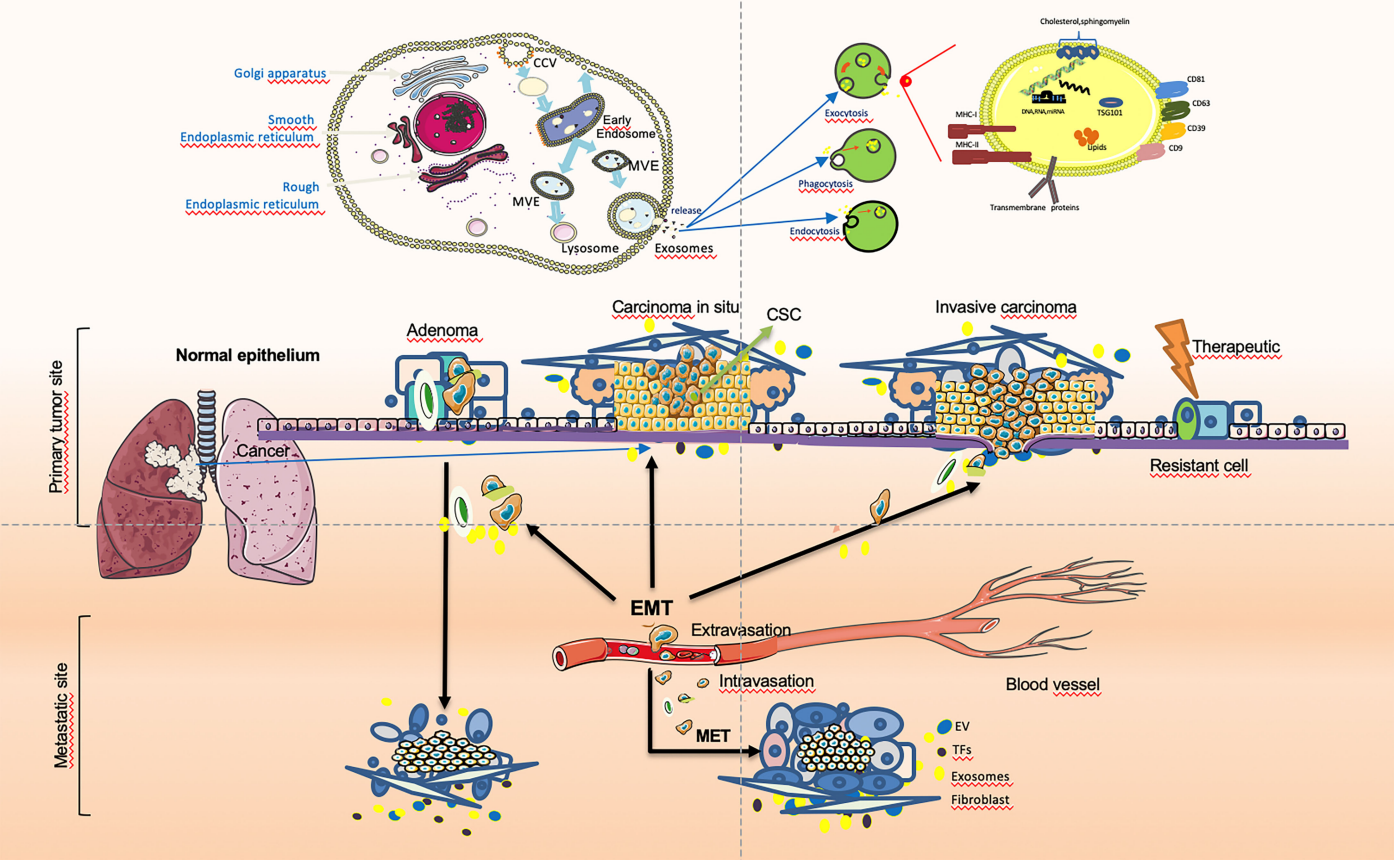
Cancer cells especially cancer stem cells(CSCs)-derived exosome-mediated EMT during cancer progression. In tumor cells, epithelial to mesenchymal transition (EMT) and transcription factors (EMT-TFs), especially tumor-derived exosomes, assign stem cell (SC) characteristics to dedifferentiated tumor cells that are called cancer stem cells (CSCs). The dissemination of tumor cells in the primary tumor and migration after the breakdown of the basement membrane (BM) can be achieved That cells initiates invasion when integrated necessary genetic aberrations and simulated by regarding signals from exosome origins at the tumor. Based on this, providing signals and maintaining the mesenchymal state of metastatic cells may be a positive contribution of EMT.EMT features may promote resistance in the period of anti-tumor therapy, leading to recurrence and poor prognosis. The imbalance between relevant regulatory networks and activated oncogenic approaches at different cancer stages may determine the extent of EMT. In particular CSC in tumor cells-secreted exosomes play an important role as messengers in the dynamic transition between EMT and MET in tumor cells. Cancer cells can activate transfer between EMT and MET while cancer cell invasion.

Except for partial activation, the EMT of tumor cells is usually reversibly activated, and cancer cells can be restored to the epithelial state through the MET during tumor progression. During cancer invasion, tumor cells undergo a dynamic transition between epithelial mesenchymalization and mesenchymal epithelialization (133). Tumor stem cell-secreted exosomes play an important role as messengers in the dynamic transition between EMT and MET in tumor cells. Cancer cells can flexibly activate EMT and MET during cancer cell invasion. The MET is synergistically induced by a variety of microenvironmental signals, which can lead to a decrease in the expression of the epithelial cell marker E-cadherin and an increase in the expression of mesenchymal cell proteins, leading to the loss of adhesion junctions and epithelial polarity in epithelial cells and the acquired invasive mesenchymal phenotype. This is the reverse process of transformation of epithelial cells into mesenchymal cells. In addition, apart from morphological changes, some cancer cells also induce the initial state of the CSC during the EMT process. The reversibility and plasticity of MET and epithelial mesenchymal stem cells are crucial. The reverse process in embryogenesis and cancer progression (MET) is critical for the final developmental cell differentiation and post-metastatic clonal growth (128, 134). Signal transmission by exosomes between tumors in the microenvironment plays an important role in the occurrence and development of tumors. The cancer cells especially CSCs-derived exosome mediated invasion and metastasis by EMT and MET during cancer progression are shown in **Figure 1**.

### CSC Exosomes Regulate the Dynamic Balance of the Tumor Niche

The differences between CSCs and non-CSCs can be attributed to the biological process of EMT (135). EMT activation requires intercellular signal transmission in normal cells and tumor cells. Signal transmission in the extracellular matrix is mainly carried out in the form of secretions, which are released by various types of cells and interact with nearby epithelial cells, leading to the intracellular signal cascade transmission of epithelial cells. These pathways lead to the expression of transcripts that coordinate the EMT expression and regulate the expression of various target genes, which finally cause the expression of mesenchymal cell characteristics.

**Figure 2:**
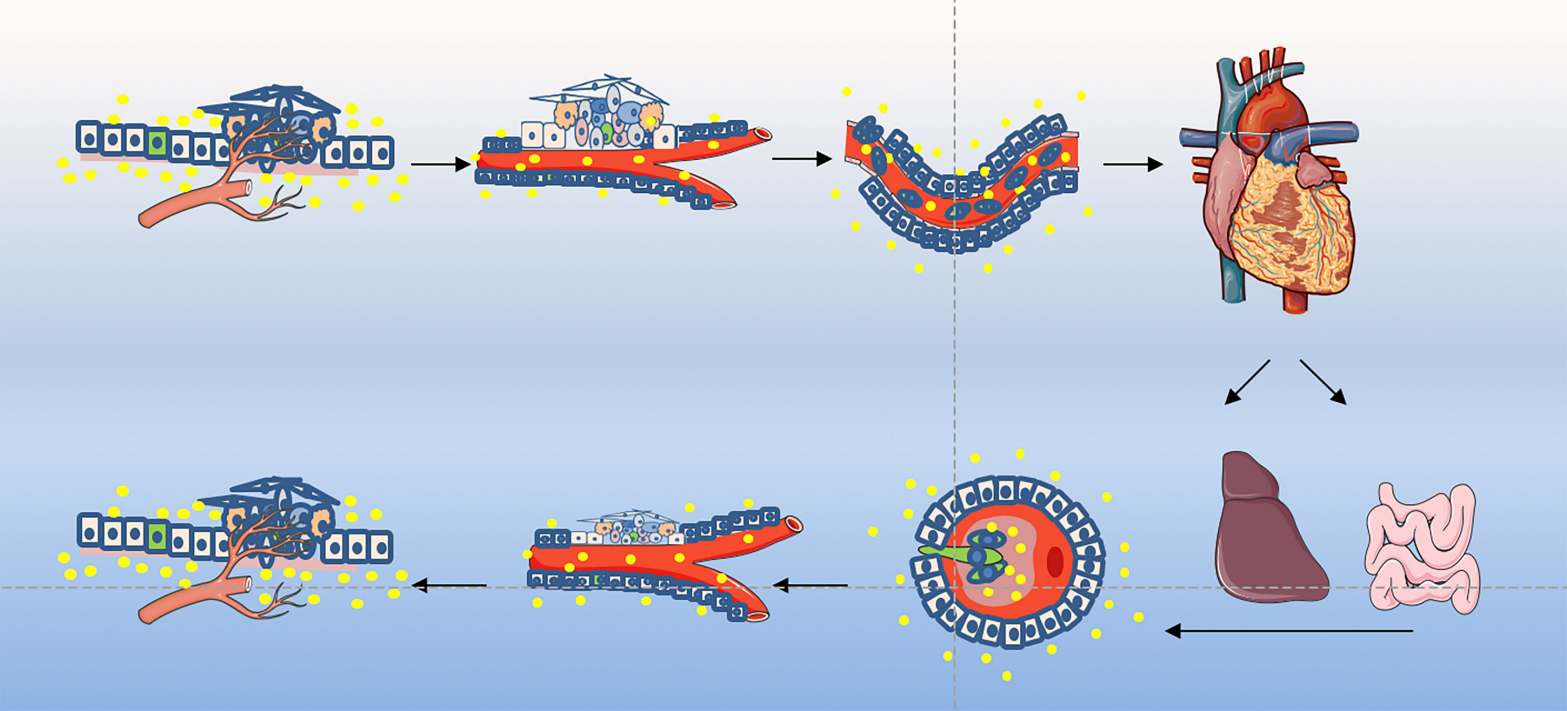
Exosome play an important role in tumor invasion–metastasis cascade. This depiction of the invasion–metastasis cascade ascribes distinct steps to the overall process. The initial step in local invasion is for the *in situ* cancer cells to break through the basement membrane. They may then infiltrate into the lymphatic vessels or microvasculature. The latter may then transport these cancer cells through the systemic circulation to distant anatomic sites, where they may become trapped and subsequently exude, forming dormant micrometastases. Some micrometastases may eventually acquire the ability to colonize the tissue in which they land, allowing them to form macroscopic metastases. The final step - colonization - appears to be the least efficient of all. The little of successfully completing all steps of this cascade process explains why the likelihood of any individual cancer cell leaving the primary tumor and becoming the founder of a distant macroscopic metastasis is low. More suggestions proposed an alternative description involving two main stages: the first enables the physical dissemination of cancer cells from the core of the primary tumor to the parenchyma of a distant tissue, while the second—colonization—depends on the adaptation of disseminated cancer cells to the microenvironment of this tissue. Exosomes as an essential part taking into the messages during the whole process.

The EMT process contributes to the progression of many cancer types (94, 136, 137). Cancer cells lose epithelial characteristics, gain invasive characteristics and stem cell-like characteristics, and also have epithelial cells that acquire stem-like properties (95). Epithelial cells may enter the stem cell state, mesenchymal state, or both states dynamically, depending on the EMT-TF process. Normal stem cells and CSCs produced in epithelial tissues usually show a mixture of epithelial and mesenchymal features, indicating that the process can only be partially promoted. MET enables cells to restore their ability to grow cohesively, thereby enabling them to colonize the site of secondary tumors. Therefore, MET is necessary for the late stage of tumor cell metastasis and colonization (138). EMT occurs in a gradual manner, showing intermediate morphological and epigenetic characteristics between epithelial and mesenchymal cells (139). However, cells in the mixed epithelial/mesenchymal phenotype tend to display the characteristics of epithelial cells and mesenchymal cells, and these hybrid epithelial/mesenchymal phenotypes are related to the collective cell migration of cancer cells (140). Exosomes secreted by CSCs play an important messenger role in this process and participate in the regulation of this transformation. The exosomes derived from cancer cells mediated invasion and metastasis play an important role during cancer progression are shown in **Figure 2**.

## Application Prospects of CSC Exosomes in Cancer

The biological behavior of cancer cell invasion and metastasis is the same as that of other biological phenotypes, which are triggered by gene function. The propensity for metastasis is related to the expression patterns of specific genes in human breast cancer. Most cells in the primary tumor show this characteristic gene expression profile (141). In other words, during the multi-stage tumor progression, the primary tumor continues to accumulate metastatic potential, and the tumor as a whole shows a characteristic expression pattern of metastasis.

In recent years there has been an increasing number of studies on the biological function of exosomes. Exosomes originate from various types of cellular secretion and are present in almost all body fluids. As a vehicle for intercellular communication, they can play an essential role in the physiological and pathological course of disease. As exosomes can be encapsulated in various contents, including lipids, proteins and RNA, they can signal and drive metastasis to specific recipient cells or tissues and organs. These exosomes, which are released into the tumor microenvironment, are received by the recipient cells, thereby inducing intracellular signaling and altered recipient cell physiology. Exosomes can occur organ-specific and direct organogenic metastasis of different metastatic tumors before tumor cells, especially tumor stem cells, reach the target organ. Exosomes are particularly important for supporting the formation of distal organogenic pre-metastatic niches. Cancer is the most common type of human tumor. The malignant epithelial cells in the tumor need to undergo EMT to acquire the ability to invade and metastasize. By blocking the expression of key regulatory factors, EMT plays an important role in the genesis of tumor EMT, MET, CSCs, and tumor metastasis to promote tumor proliferation, invasion, adhesion, angiogenesis, and acquired drug resistance, thereby affecting the process of tumor development. Metastatic dormancy is a challenge in tumor research; for instance, in breast cancer, metastases are found decades after the removal of the primary tumor, due to the long incubation period and multiple micro-metastases occurring.

Exosomes secreted by tumor stem cells are regulated through the tumor stem cell-tumor microenvironment-target tissue interface, potentially serving as metastasis markers and promising as entry points for blocking tumor metastasis.

The study of tumor stem cell exosomes facilitates the in-depth understanding of the mechanism of metastasis, which will hopefully lead to new treatment avenues. CSC exosomes, which are important in the promotion of CSCs and tumor foci, the effect of primary tumors and EMT, and the recurrence of metastases and cancer, have important research value for exploring cancer progression and tumor treatment. The personalized and pinpoint treatment of anti-tumors further raises the requirement to diagnose the mechanisms of tumor metastasis and recurrence. The close relationship between exosomes and metastasis is predicted to be a diagnostic marker for tumor recurrence; targeting the signal transduction of exosomes for interventional blockade will likely be useful for clinical application of therapeutic new therapies; the targeting ability of exosomes can also be utilized to target the contents of exosomes as drug carriers for research, which is expected to target drug delivery for effective treatment of recurrent tumors. The in-depth study of the effect of tumor stem cell exosomes on the regulation of tumor metastasis and recurrence in the process of cancer has far-reaching significance for the precise diagnosis of anti-tumor phased treatment, prognosis judgment, and other fields.

## Author Contributions

KL conceptualized and wrote the article. XG, BK, DW, and YW revised the article. YW revised and finalized the article. All authors contributed to the article and approved the submitted version.

## Funding

Project supported by Jilin Province Science and Technology Support Program [Grant number 20200404121YY]; Education Department of Jilin Province [Grant number JJKH20201122KJ]; Guangzhou Science and Technology Planning Project, China (201904010443).

## Conflict of Interest

The authors declare that the research was conducted in the absence of any commercial or financial relationships that could be construed as a potential conflict of interest.

## Publisher’s Note

All claims expressed in this article are solely those of the authors and do not necessarily represent those of their affiliated organizations, or those of the publisher, the editors and the reviewers. Any product that may be evaluated in this article, or claim that may be made by its manufacturer, is not guaranteed or endorsed by the publisher.

## Abbreviations

CSCs, cancer stem cells; EMT, endothelial-mesenchymal transition; EVs, extracellular vesicles; ILVs, intraluminal vesicles; MET, mesenchymal-epithelial transformation; miRNAs, microRNAs; MVBs, multivesicular bodies; MVEs, multivesicular endosomes; TME, tumor microenvironment.
